# Leveraging diffusion models for unsupervised out-of-distribution detection on image manifold

**DOI:** 10.3389/frai.2024.1255566

**Published:** 2024-05-09

**Authors:** Zhenzhen Liu, Jin Peng Zhou, Kilian Q. Weinberger

**Affiliations:** Department of Computer Science, Cornell University, Ithaca, NY, United States

**Keywords:** out-of-distribution detection, diffusion models, score-based models, generative modeling, manifold learning

## Abstract

Out-of-distribution (OOD) detection is crucial for enhancing the reliability of machine learning models when confronted with data that differ from their training distribution. In the image domain, we hypothesize that images inhabit manifolds defined by latent properties such as color, position, and shape. Leveraging this intuition, we propose a novel approach to OOD detection using a diffusion model to discern images that deviate from the in-domain distribution. Our method involves training a diffusion model using in-domain images. At inference time, we lift an image from its original manifold using a masking process, and then apply a diffusion model to map it towards the in-domain manifold. We measure the distance between the original and mapped images, and identify those with a large distance as OOD. Our experiments encompass comprehensive evaluation across various datasets characterized by differences in color, semantics, and resolution. Our method demonstrates strong and consistent performance in detecting OOD images across the tested datasets, highlighting its effectiveness in handling images with diverse characteristics. Additionally, ablation studies confirm the significant contribution of each component in our framework to the overall performance.

## 1 Introduction

The goal of out-of-distribution (OOD) detection is to ascertain if a given data point comes from a specific domain. This task is crucial given that machine learning models generally require that the distribution of test data mirrors the distribution of the training data. In cases where the test data deviates from the training distribution, the models can generate meaningless or deceptive results. This could be especially harmful for tasks in high-stake areas like healthcare (Hamet and Tremblay, 2017) and criminal justice (Rigano, 2019).

The OOD detection task has been examined under settings with access to varied amount of information. These settings can be categorized as supervised and unsupervised. Among supervised settings, the most informed scenario makes the assumption that exemplar out-of-domain data are available. One can then incorporate them in the training of neural networks to enhance their ability to recognize out-of-domain inputs (Hendrycks et al., 2018; Ruff et al., 2019). Various methods excel on identifying out-of-domain data when that resemble the training examples, but their performance deteriorates on out-of-domain inputs that are not represented in the training process. In practical applications, inputs are often highly diverse, and it is challenging to construct a truly representative set of out-of-domain examples. A more feasible setting is to only leverage in-domain classifiers or class labels (Hendrycks and Gimpel, 2016; Liang et al., 2017; Lee et al., 2018; Huang et al., 2021; Wang et al., 2022). Although this setting is less restrictive, it still requires two essential conditions: well-defined categorization of the in-domain data and an adequate amount of labeled data. These conditions do not hold for many tasks. In contrast, the fully unsupervised setting only require access to unlabeled in-domain data, which can often be obtained with low cost and in abundant quantities. As a result, it is ideal to develop OOD detectors under the fully unsupervised setting.

Recently, the diffusion models (DMs), a type of generative models, have received increasing attention in the machine learning community (Ho et al., 2020; Song et al., 2020). DMs operate on two procedures: The forward operation performs iterative noise addition to an image's pixels and transforms it into a sample drawn from a noise distribution. The backward operation—performed by a dedicated neural network—gradually removes noise from the image, guiding a noise image toward a specific image manifold.

In this paper, we show that we can leverage DMs as a mapping to a manifold, and use it for unsupervised OOD detection. Conceptually, if an image is lifted from its manifold, a diffusion model trained over the same manifold can guide it back to its original manifold. However, if the diffusion model has been trained on a different manifold, it would lead the lifted image toward its own training manifold, resulting in a substantial distance between the original and the mapped images. Therefore, we can identify out-of-domain images based on this distance.

**Figure 1:**
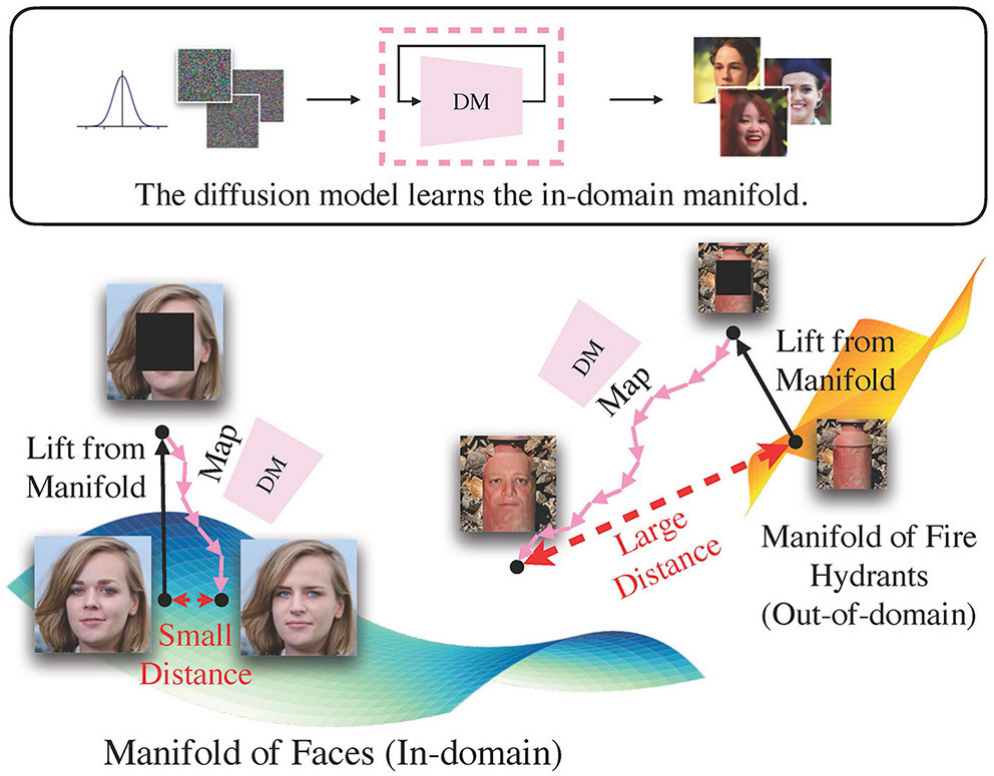
The intuition behind LMD. In essence, LMD leverages a diffusion model as a mapping toward the in-domain manifold. It applies a mask to the image to lift it from its original manifold, and uses the diffusion model to guide it toward the in-domain manifold. If an image is in-domain, it would generally have smaller distance between the original and mapped locations than out-of-domain images.

To this end, we introduce an innovative unsupervised method for out-of-distribution detection, **L**ift, **M**ap, **D**etect (LMD), that embodies the aforementioned concept. **Lifting** is performed through image corruption. For instance, a face image that has been masked in the center will no longer fit into the face image category. Previous research by Song et al. (2020) and Lugmayr et al. (2022) have demonstrated that the diffusion model can perform inpainting, i.e., restoring missing areas in an image with visually convincing content, without the need for additional training. This allows us to **map** the mapped image via inpainting with a in-domain diffusion model. We can employ a conventional image similarity metric to calculate the distance between the original and mapped images, and **detect** an out-of-domain image when there is a significant distance. In Figure 1, we provide an example: A diffusion model trained with face images maps a lifted in-domain face image closer to its original location, while moving an lifted fire hydrant, an out-of-domain image, further away.

Our main contributions include: (1) We propose an innovative unsupervised OOD detection technique, *Lift, Map, Detect* (LMD), that utilizes of the inherent manifold mapping capacity of diffusion models, and incorporates design choices that enhance the distinguishability between in-domain and out-of-domain data. (2) We conduct extensive experiments on various image datasets with different characteristics to illustrate the versatility of LMD. (3) We present in-depth analysis, visualizations and ablations to confirm LMD's underlying hypothesis and provide insights into LMD's behaviors.

## 2 Materials and methods

### 2.1 Preliminaries

**Problem formulation**. Formally, we define the unsupervised out-of-distribution (OOD) task as follows: We aim to build a detector to identify data points **x** that deviate from a distribution of interest D. The detector should be built using only unlabeled data **x_1_**, ⋯ , **x_n_** sampled from D. It should assign an OOD score *s*(**x**) that positively correlates with the likelihood of **x** *not* belonging to D.

**Diffusion models**. In this section, we present a brief summary of the concepts behind the diffusion model (DM). It is a class of generative models that can learn complex distributions. It involves a forward process of diffusion and a backward process of denoising. Diffusion corrupts the original data with noise, while denoising—performed by a learned neural network—progressively reduces noise from the corrupted image. There are various formulations of diffusion models, such as score-based generative models (Song and Ermon, 2019) and stochastic differential equations (Song et al., 2020). A comprehensive review can be found in Yang et al. (2022).

LMD is agnostic to the different DM variants. Here, we describe one prominent variant: the Denoising Diffusion Probabilistic Models (DDPMs) (Sohl-Dickstein et al., 2015; Ho et al., 2020). DDPM's diffusion process begins with a data sample *x*_0_, and injects Gaussian noise at every subsequent step *t* = 1, 2, ⋯ , *T* following Equation (1)


(1) $$\begin{array}{l} q\left(x_{t}|x_{t-1}\right)=N\left(x_{t};\sqrt{1-\beta_{t}}x_{t},\beta_{t}\text{I}\right) \end{array}$$

where β_*t*_ adheres to a predetermined variance schedule. The denoising process has a prior distribution $x_{T}\sim N\left(0,1\right)$, and formulates the process following Equation (2)


(2) $$\begin{array}{l} p_{\theta }\left(x_{t-1}|x_{t}\right)=N\left(x_{t-1};\mu_{\theta }\left(x_{t},t\right),\Sigma_{\theta }\left(x_{t},t\right)\right) \end{array}$$

where both μ_θ_(*x*_*t*_, *t*) and Σ_θ_(*x*_*t*_, *t*) are parametrized by a neural network θ.

### 2.2 Lift, Map, Detect

**Figure 2:**
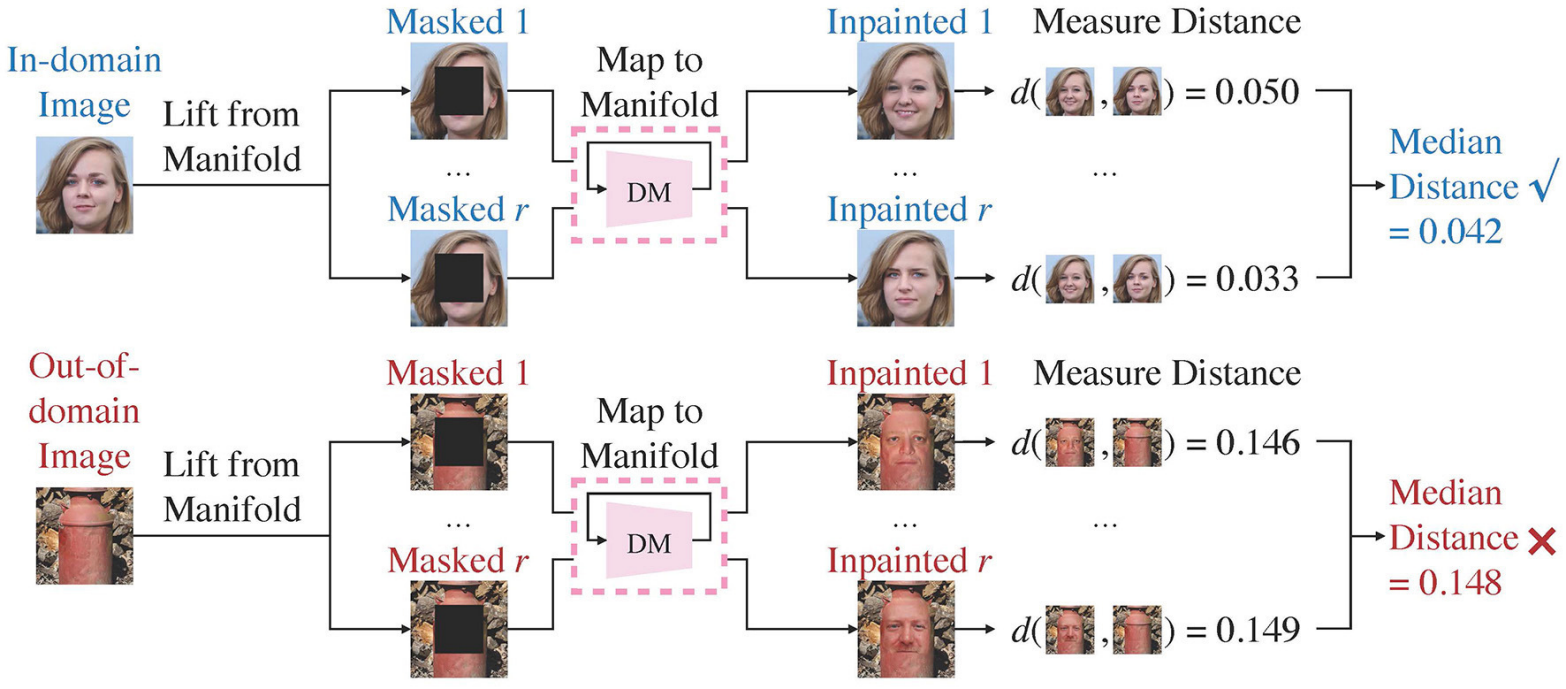
Overview of the LMD process. LMD utilizes a diffusion model trained over the in-domain manifold. It repeatedly lifts an image from its manifold by masking, and maps it toward the diffusion model's training manifold by inpainting. It measures the median distance between the original and the mapped images, and considers images with larger distance as out-of-domain.

Lift, Map, Detect (LMD) is inspired by the observation that a diffusion model maps images toward the manifold it is trained on. Concretely, it leverages a diffusion model trained over unlabeled in-domain data. Given a test image, LMD applies corruption techniques to lift it from its original manifold, and utilizes the diffusion model to map it toward the in-domain manifold on which the model is trained. As depicted in Figure 1, if the image is indeed in-domain, the model can map it back to its manifold close to its original location. Conversely, if the image belongs to a different manifold, then the diffusion model would redirect it toward the in-domain manifold, moving it further away from its original location. Hence, out-of-domain images often have larger distance between the original and mapped images than in-domain images, and LMD identifies images with large distance as OOD. Figure 2 presents the general framework of LMD in Figure 2, and Algorithm 1 provides a succinct representation of the LMD algorithm. Subsequent sections explain each component of LMD in detail.

**Algorithm 1:**
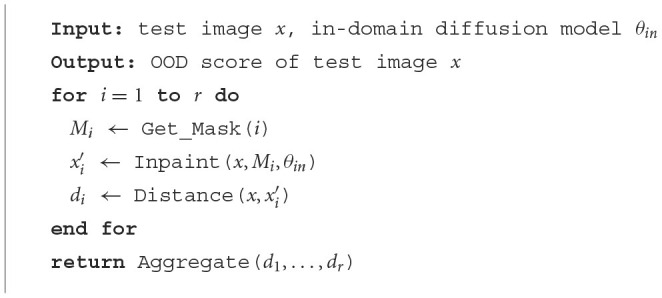
Lift, Map, Detect (LMD).

#### 2.2.1 Lifting and mapping images

LMD lifts an image by masking parts of it, and maps it by inpainting over the masked area. For convenience, we also refer the lifted and mapped images as masked and reconstructed images, respectively. Masking provides a straightward way of controlling the extent to which an image is lifted, as larger masked area generally corresponds to larger deviation from the manifold. Furthermore, recent studies have shown that vanilla diffusion models can perform inpainting without the need for retraining, regardless of the size or shape of the masked regions. This highlights masking and inpainting as an intuitive strategy. Algorithm 2 describes the high-level process of inpainting with diffusion models. Additionally, we observe that an alternative way of lifting and mapping an image is to just add noise to it and then denoise with the diffusion model. We compare this instantiation with masking and inpainting in Table 4.

**Algorithm 2:**
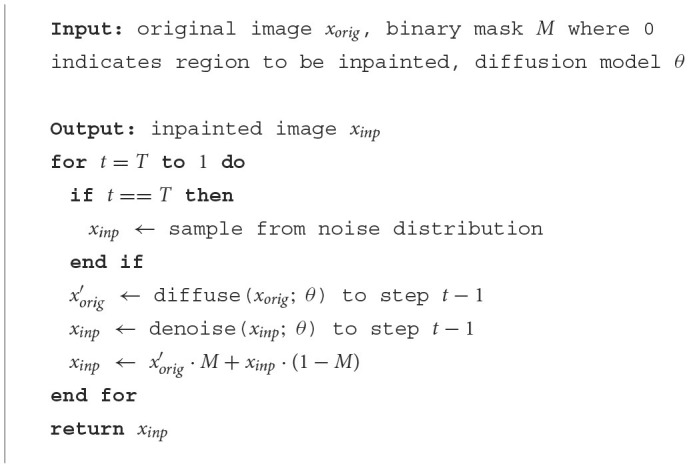
Inpaint.

LMD operates based on the assumption that in-domain images have smaller reconstruction distance than out-of-domain images. In practice, the validity of this assumption depends on two factors. First of all, inpainting with a diffusion model is stochastic. This occasionally leads to unfaithful in-domain reconstructions or faithful out-of-domain reconstructions. Consequently, a single reconstruction distance provides a noisy signal for identifying OOD images. To mitigate the randomness, we perform **multiple reconstructions** for each image, and use the median reconstruction distance as the OOD score. Our experiments in Section 3.4.3 show that this can significantly improve the detection performance.

Another factor to consider is the amount of information removed from an image. In the extreme case where the whole image is masked out, the reconstruction would be a random image from the in-domain manifold. This could lead to large reconstruction distance for both in-domain and out-of-domain images, especially when the in-domain distribution is diverse. Conversely, if only one pixel is removed from an image, then both in-domain and out-of-domain reconstructions would be highly faithful. Therefore, a mask should ideally provide sufficient clues for the diffusion model to map a lifted in-domain image close to its original location, while creating enough space to produce dissimilar out-of-domain reconstructions.

**Figure 3:**
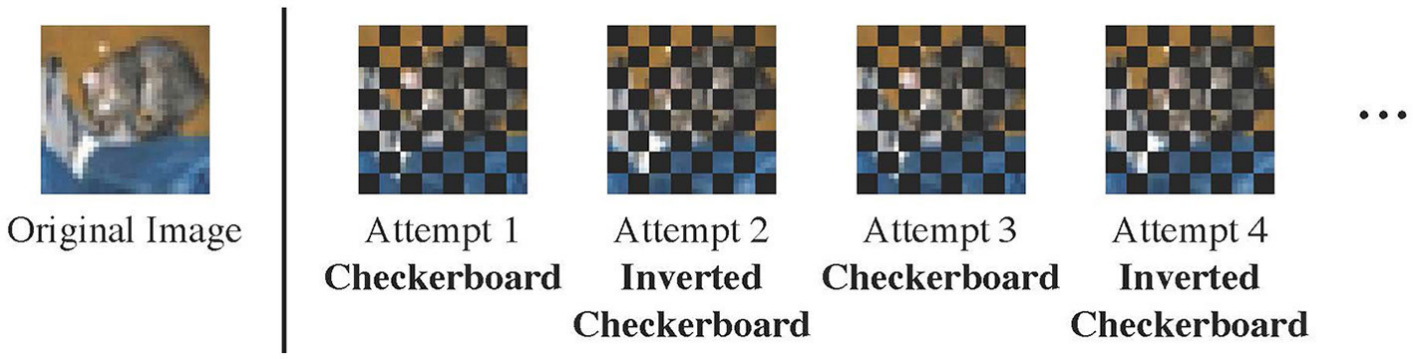
The alternating checkerboard mask. We flip the masked and unmasked regions at each reconstruction attempt. The example in the figure is 8 × 8.

In this regard, we propose to use the **alternating checkerboard** ***N***×***N*** mask (Figure 3). For simplicity, we assume that images are square-shaped with size *L*×*L*; extension to rectangular-shaped images is straightforward. The checkerboard mask divides the image into an *N*×*N* grid of patches, where each patch has size $\frac{L}{N}\times \frac{L}{N}$. It masks out every other patch in a checkerboard-like fashion, covering 50% of an image in total. During multiple reconstructions, the masked and unmasked patches are flipped at each reconstruction attempt. This ensures that salient characteristics of an out-of-domain images are covered at some attempts. We default to *N* = 8. Experiments with different values of *N* can be found in Table 2.

#### 2.2.2 Measuring reconstruction distance

We use the Learned Perceptual Image Patch Similarity (LPIPS) (Zhang et al., 2018) metric to measure the distance between the original and reconstructed images. LPIPS utilizes calibrated intermediate activations of a pretrained neural network as features, and measures the normalized ℓ_2_ distance between the features of two images. This yields a value between 0 and 1, where lower value indicates higher similarity. We employ the version with AlexNet (Krizhevsky et al., 2012) backbone pretrained on ImageNet.^1^ LPIPS has been observed to align with human perception of image similarity (Zhang et al., 2018), and has been applied in research on a wide range of tasks (Karras et al., 2019; Alaluf et al., 2021; Meng et al., 2021) and image modalities (Gong et al., 2021; Lugmayr et al., 2022; Toda et al., 2022). Experiments with alternative metric choices in Table 3.

## 3 Results

### 3.1 Experiment settings

We benchmark LMD against existing unsupervised OOD detection methods on widely used datasets. We provide fine-grained analysis and visualizations of the reconstructed images to better understand LMD's performance. Additionally, we perform ablation studies to analyze the individual components of LMD.

#### 3.1.1 Baselines

We compare LMD with seven existing baselines, covering three mainstream classes of methods: likelihood-based, reconstruction-based and feature-based. For likelihood-based methods, we consider Likelihood **(Likelihood)** (Bishop, 1994), Input Complexity **(IC)** (Serrà et al., 2019) and Likelihood Regret **(LR)** (Xiao et al., 2020). We obtain the likelihood from the diffusion model using Song et al. (2020)'s approach.^2^ We adapt the official GitHub repository of Likelihood Regret^3^ for both Likelihood Regret and Input Complexity. For Input Complexity, we leverage the likelihood from the diffusion model to ensure fairness in comparison; we have experimented with both the PNG compressor and the JPEG compressor, and we report the results from the PNG compressor due to its superior performance. For reconstruction-based methods, we consider Reconstruction with Autoencoder and Mean Squared Error loss **(AE-MSE)**, AutoMahalanobis **(AE-MH)** (Denouden et al., 2018) and AnoGAN **(AnoGAN)** (Schlegl et al., 2017). For feature-based method, we consider Pretrained Feature Extractor + Mahalanobis Distance **(Pretrained)** (Xiao et al., 2021). We use our own implementation as we did not find any existing implementation to our best efforts.

#### 3.1.2 Evaluation

We evaluate the performance of LMD and the baselines using the area under Receiver Operating Characteristic curve (ROC-AUC), following the practice of existing works (Hendrycks and Gimpel, 2016; Ren et al., 2019; Xiao et al., 2021). OOD detection methods commonly produce numeric OOD scores, and apply a decision threshold to classify data as in-domain or out-of-domain. The ROC curve plots the true positive rate against the false positive rate at various decision thresholds, and ROC-AUC measures the area under the curve. ROC-AUC ranges between 0 and 1, with higher values indicating better performance. A detector achieves ROC-AUC >0.5 when it in general assigns higher OOD scores to out-of-domain images than in-domain images. Conversely, it yields ROC-AUC < 0.5 when it in general assigns higher OOD scores for in-domain images.

#### 3.1.3 Datasets

For quantitative evaluations, we consider pairwise combinations of CIFAR10 (Krizhevsky, 2009), CIFAR100 (Krizhevsky, 2009) and SVHN (Netzer et al., 2011), and pairwise combinations of MNIST (LeCun et al., 2010), KMNIST (Clanuwat et al., 2018), and FashionMNIST (Xiao et al., 2017), as the in-domain and out-of-domain datasets. This yields 12 pairs in total. For qualitative evaluations, we further present visualizations on two pairs of in-domain vs. out-of-domain datasets with higher image resolutions: CelebA-HQ (Karras et al., 2017) vs. ImageNet (Russakovsky et al., 2015), and LSUN bedroom (Yu et al., 2015) vs. LSUN classroom (Yu et al., 2015). We standardize these images to 256 × 256.

#### 3.1.4 Implementation details of LMD

We build LMD on top of Song et al. (2020)'s implementation. For datasets in Table 3, we use DDPM++ models with SubVP SDE. We take Song et al. (2020)'s pretrained CIFAR10 checkpoint, and train from scratch for the other datasets. We use alternating checkerboard 8 × 8 mask (Figure 3), reconstruction distance metric LPIPS and 10 reconstructions per image for LMD.

For the higher resolution datasets, we use NCSN++ models with VE SDE. We take Song et al. (2020)'s pretrained FFHQ (Karras et al., 2019) checkpoint for CelebA-HQ vs. ImageNet. This is to avoid model memorization concerns given that the CelebA-HQ checkpoint is pretrained over the whole dataset. We use Song et al. (2020)'s pretrained LSUN bedroom checkpoint for LSUN bedroom vs. LSUN classroom. For these datasets, we consider a checkerboard 4 × 4 mask, a checkerboard 8 × 8 mask and a square-centered mask, with *one* reconstruction per image. We additionally report the ROC-AUC from our default configuration of alternating 8 × 8 checkerboard and 10 reconstructions per image as a reference. We use LPIPS as the distance metric.

### 3.2 Quantitative results and analysis

**Figure 4:**
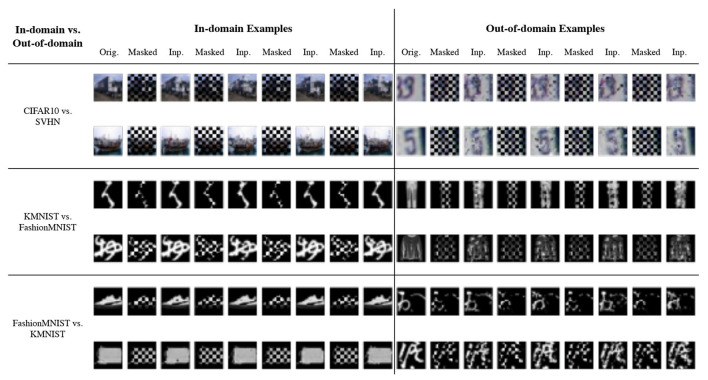
Example reconstructions from three pairs of dataset. “Orig.” is the original image and “Inp.” is the inpainted image. Generally, the in-domain reconstructions are faithful while the out-of-domain reconstructions are noisy and dissimilar.

We present the OOD detection performance of LMD and the baselines on 12 dataset pairs in Table 1. LMD attains the highest ROC-AUC on five pairs, while demonstrating consistent and strong performance on others. Specifically, on CIFAR100 vs. SVHN, it attains 10% higher ROC-AUC than the best baseline performance. LMD also attains the highest average ROC-AUC of 0.907, which is 9% higher than the best average performance among the baselines. We visualize examples of the in-domain and out-of-domain reconstructions of LMD in Figure 4. In general, in-domain reconstructions resemble their original images, while out-of-domain reconstructions are fragmented and noisy.

**Table 1:** ROC-AUC of LMD and the baselines.

**ID**	**OOD**	**Likelihood**	**IC**	**LR**	**Pretrained**	**AE-MSE**	**AE-MH**	**AnoGAN**	**LMD**
CIFAR10	CIFAR100	0.520	0.568	0.546	**0.806**	0.510	0.488	0.518	0.607
	SVHN	0.180	0.870	0.904	0.888	0.025	0.073	0.120	**0.992**
CIFAR100	CIFAR10	0.495	0.468	0.484	0.543	0.509	0.486	0.510	**0.568**
	SVHN	0.193	0.792	0.896	0.776	0.027	0.122	0.131	**0.985**
SVHN	CIFAR10	0.974	0.973	0.805	**0.999**	0.981	0.966	0.967	0.914
	CIFAR100	0.970	0.976	0.821	**0.999**	0.980	0.966	0.962	0.876
MNIST	KMNIST	0.948	0.903	0.999	0.887	0.999	**1.000**	0.933	0.984
	FashionMNIST	0.997	**1.000**	0.999	0.999	**1.000**	**1.000**	0.992	0.999
KMNIST	MNIST	0.152	0.951	0.431	0.582	0.102	0.217	0.317	**0.978**
	FashionMNIST	0.833	**0.999**	0.557	0.993	0.896	0.868	0.701	0.993
FashionMNIST	MNIST	0.172	0.912	0.971	0.647	0.804	0.969	0.835	**0.992**
	KMNIST	0.542	0.584	0.994	0.730	0.976	**0.996**	0.912	0.990
Average		0.581	0.833	0.783	0.821	0.651	0.679	0.658	**0.907**

Higher value is better. We use the default configuration of alternating checkerboard 8 × 8, LPIPS metric and 10 reconstructions per image for all experiments. LMD consistently demonstrates strong performance and attains the highest average ROC-AUC. The bold values mean the best performance, i.e., the highest ROC-AUC, among the evaluated methods in each setting, where a setting is either an in-domain vs. out-of-domain dataset pair or the average across all dataset pairs.

**Figure 5:**
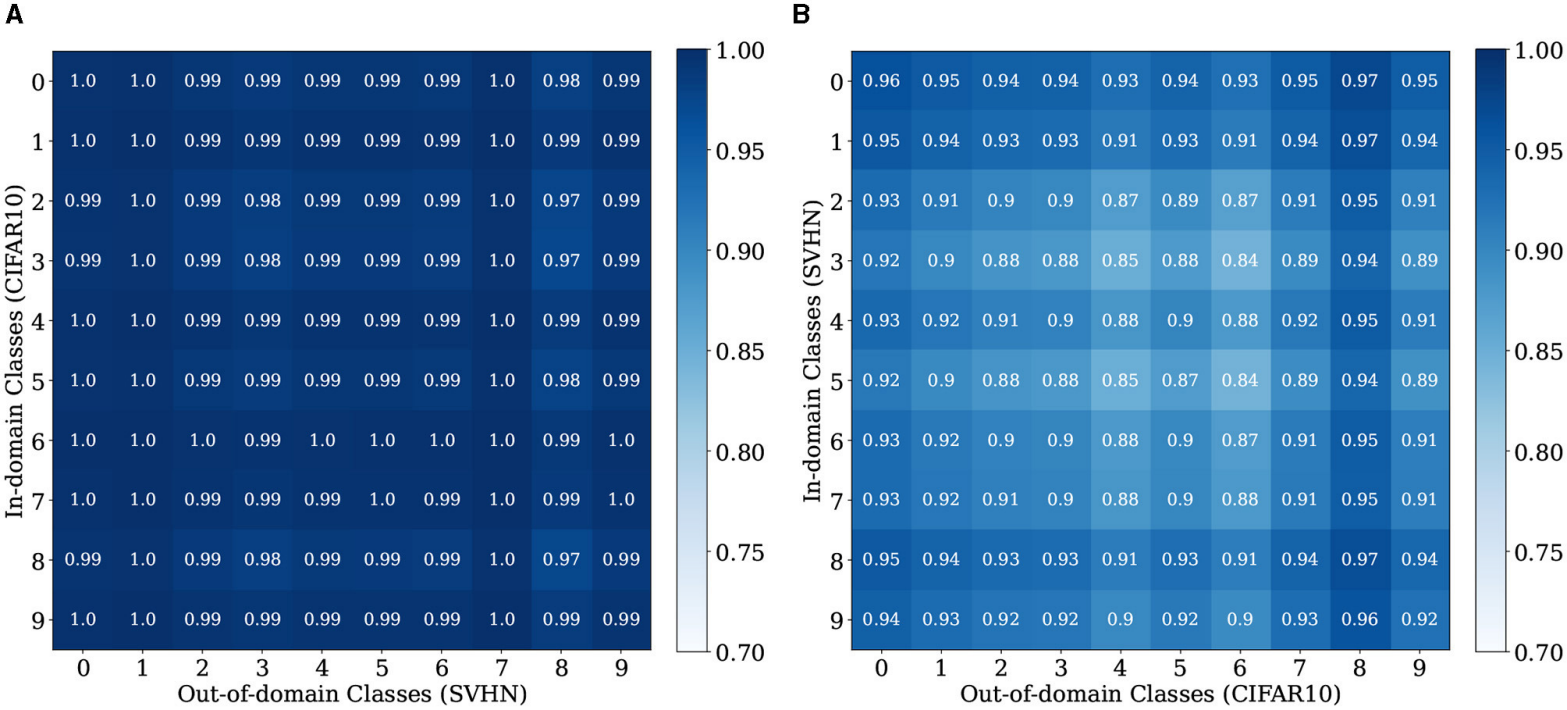
Per-Class ROC-AUC for CIFAR10 vs. SVHN and SVHN vs. CIFAR10. The classes for CIFAR10 are: 1, airplane; 2, automobile; 3, bird; 4, cat; 5, deer; 6, dog; 7, frog; 8, horse; 9, ship; and 10, truck. **(A)** CIFAR10 vs. SVHN. **(B)** SVHN vs. CIFAR10.

We further conduct fine-grained analysis to understand LMD's performance. We observe that each dataset in Table 1 consists of images from multiple distinct semantic categories, forming a diverse data distribution. For example, CIFAR10 comprises 10 different objects or animals, and SVHN comprises 10 digits. We seek to understand whether LMD performs similarly across different semantic categories, or if certain categories are more challenging for LMD than the others. Specifically, we group the images by their ground truth classes, and examine the distinguishability of the OOD scores for each pair of classes of the in-domain vs. out-of-domain datasets. We present the results for CIFAR10 vs. SVHN and SVHN vs. CIFAR10 in Figure 5. On CIFAR10 vs. SVHN, all pairs of classes are highly distinguishable, with ROC-AUC ranging from 0.97 to 1. This is unsurprising given that LMD attains strong performance of ROC-AUC 0.992 on this pair. On SVHN vs. CIFAR10, pairwise performance shows visible variation, with ROC-AUC ranging from 0.84 to 0.97. Specifically, the ROC-AUC is relatively low when the in-domain class is “3” or “5,” and when the out-of-domain class is “deer” or “frog.” This suggests that the reason behind LMD's satisfactory but suboptimal performance on SVHN vs. CIFAR10 is primarily attributed to the relative difficulty in distinguishing between some of the semantic categories.

### 3.3 Qualitative studies on higher resolution images

**Figure 6:**
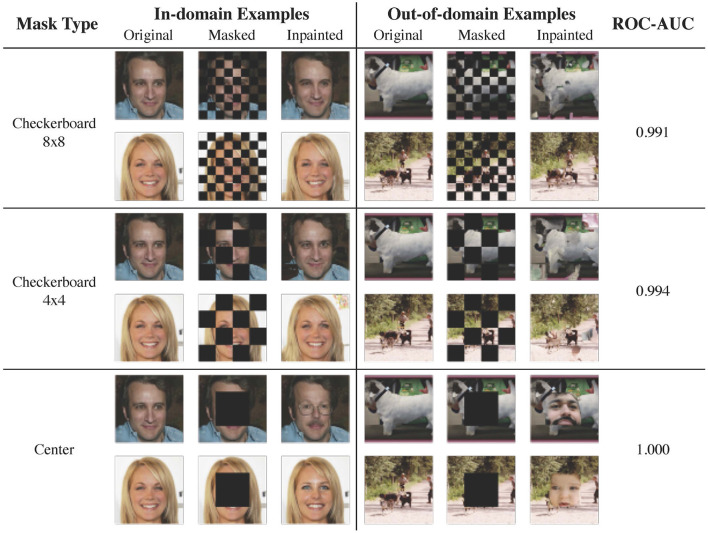
Examples of image reconstruction from CelebA-HQ (in-domain) and ImageNet (out-of-domain). For out-of-domain reconstructions, the checkerboard masks result in local inconsistencies, while the center mask hallucinates faces. In this case, employing larger masked patches slightly improves the performance.

**Figure 7:**
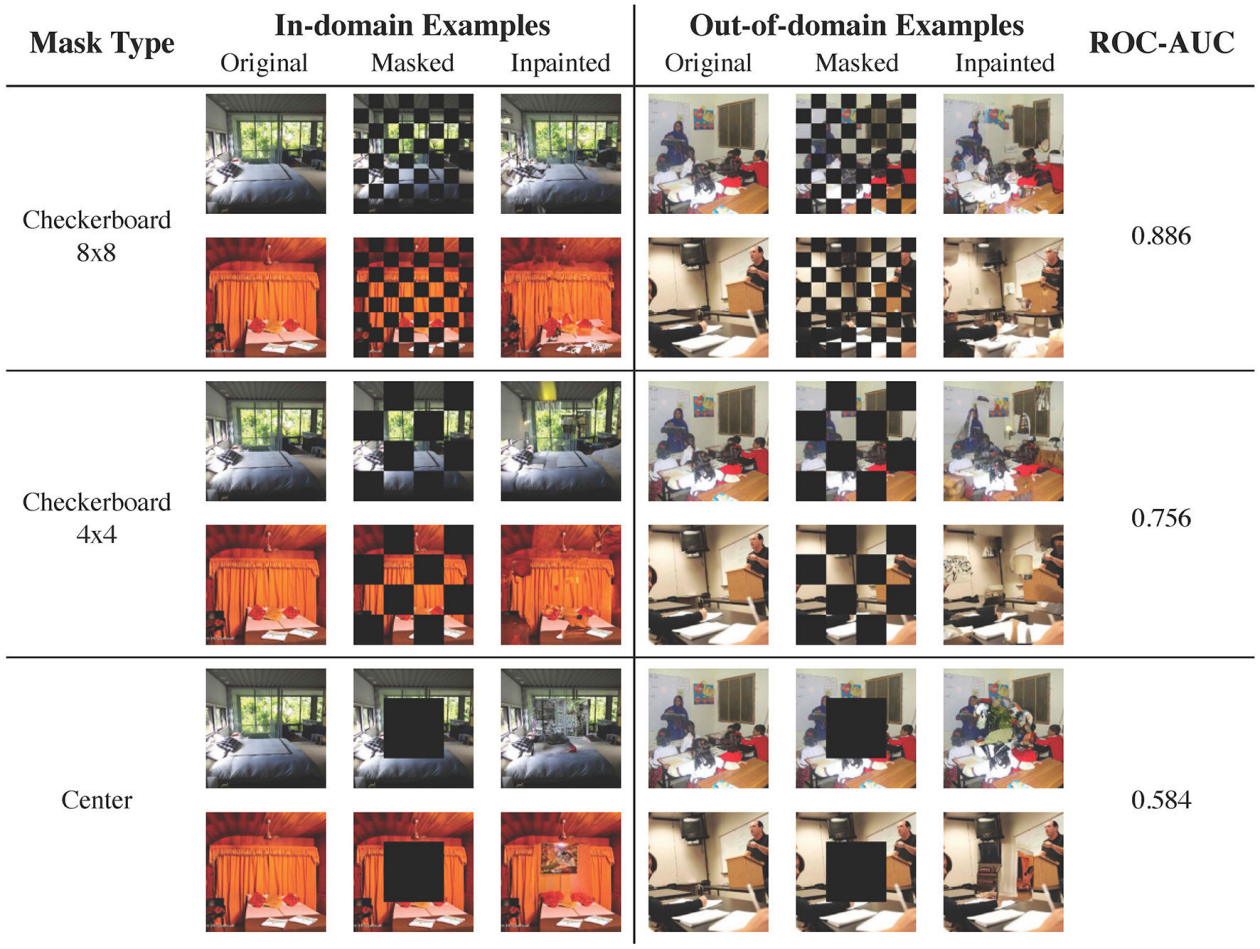
Reconstruction examples from LSUN bedroom (in-domain) and LSUN classroom (out-of-domain). As bedroom images are diverse and contain richer details, a mask with smaller patches is preferrable.

We show qualitative results on images with resolution 256 × 256 for two in-domain/out-of-domain pairs: CelebA-HQ vs. ImageNet (Figure 6) and LSUN bedroom vs. LSUN classroom (Figure 7). The ROC-AUCs in the images correspond to LMD's performance with only *one* reconstruction attempt. As a reference, under our default configuration of alternating checkerboard 8 × 8 mask and 10 reconstruction attempts, CelebA-HQ vs. ImageNet has a ROC-AUC of **0.993**, and LSUN bedroom vs. LSUN classroom has a ROC-AUC of **0.927**.

For CeleA-HQ vs. ImageNet, LMD performs competitively under all three mask choices, and achieves ROC-AUC ranging from 0.991 to 1 even without multiple reconstructions. Given the highly structured nature of human faces, the in-domain reconstructions under all three masks are accurate. For the out-of-domain images, reconstructions under the checkerboard masks contain local distortions, while reconstructions under the center mask tend to hallucinate faces. As a result, in this case, the in-domain and out-of-domain reconstructions become more discernible when employing larger patches in masking.

For LSUN bedroom vs. LSUN classroom, the checkerboard 8 × 8 mask attains strong results, while the checkerboard 4 × 4 mask and the center-squared mask demonstrate suboptimal performance. This is because bedroom images exhibit greater variation and contain more intricate details. Consequently, when large patches are masked, the diffusion model may fill in plausible yet different content, resulting in significant reconstruction discrepancies for in-domain images. In fact, even with the checkerboard 8 × 8 mask, the diffusion model may hallucinate or alter elements in the bedroom inpaintings. Moreover, the complex and diverse nature of bedroom images poses substantial challenges for the diffusion model to accurately learn the in-domain distribution; samples and inpaintings from the LSUN bedroom model generally have lower quality than those from the CelebA-HQ model.

Results from these two dataset pairs collectively demonstrate that LMD could scale to higher resolution images with richer details. They also highlight the checkerboard 8 × 8 mask as a versatile default choice, as it is effective for both structured and diverse in-domain distributions. For further discussions on mask choices, please refer to Section 3.4.1.

### 3.4 Ablation studies

#### 3.4.1 Mask choice

**Figure 8:**
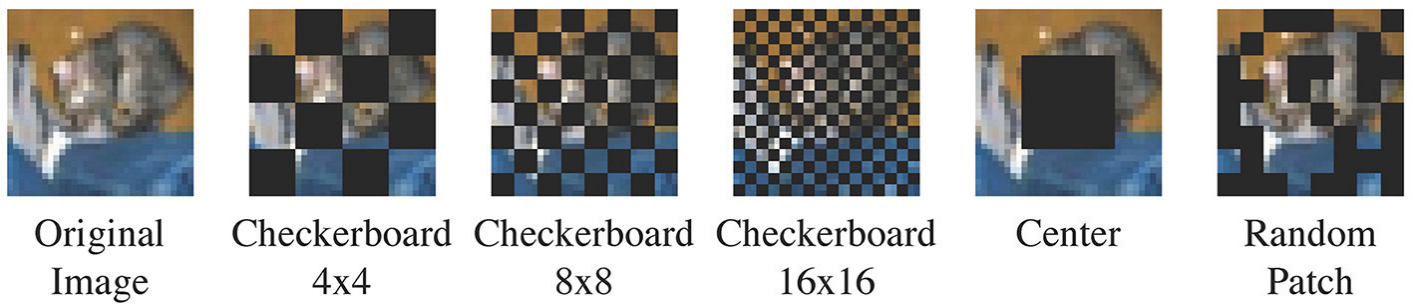
Masks used in the mask ablation. The random patch mask in the figure is just one example; a different pattern is sampled each time.

Table 2 presents the performance of LMD under alternative mask choices. Besides our default mask, we consider alternating checkerboard 4 × 4, alternating checkerboard 16 × 16, a fixed 8 × 8 checkerboard for which we do not perform the flipping operation, a square-centered mask, and a random patch mask following (Xie et al., 2022).^4^ Figure 8 visualizes the mask patterns. We experiment on three dataset pairs: CIFAR10 vs. CIFAR100, CIFAR10 vs. SVHN and MNIST vs. KMNIST. For all the mask choices, we perform 10 reconstructions per image and use LPIPS as the reconstruction distance metric.

**Table 2:** Performance of ROC-AUC on three dataset pairs with different mask types.

**Mask type**	**CIFAR10 vs. CIFAR100**	**CIFAR10 vs. SVHN**	**MNIST vs. KMNIST**
Alternating checkerboard 4 × 4	0.594	0.987	0.923
Alternating checkerboard 8 × 8	**0.607**	**0.992**	0.984
Alternating checkerboard 16 × 16	0.597	0.981	**0.997**
Fixed checkerboard 8 × 8	0.601	0.990	0.974
Center	0.570	0.978	0.479
Random patch	0.591	0.990	0.912

The alternating checkerboard 8 × 8 shows strong and consistent results. The bold values mean the best performance, i.e., the highest ROC-AUC, among the evaluated methods in each setting, where a setting is either an in-domain vs. out-of-domain dataset pair or the average across all dataset pairs.

Our default mask choice of alternating checkerboard 8 × 8 shows consistent and strong performance. Alternating checkerboard 16 × 16 mask, fixed checkerboard 8 × 8 mask and the random patch mask are competitive but underperform the default choice. Nevertheless, alternating checkerboard mask is recommended over fixed checkerboard mask or random patch mask, as it ensures that all parts of the image are covered in some of the reconstruction attempts. Alternating checkerboard 4 × 4 and square-centered masks show suboptimal performance on MNIST vs. KMNIST. This is because they mask out too much information from the images, and therefore lead to unfaithful reconstructions for both in-domain and out-of-domain images.

#### 3.4.2 Reconstruction distance metric

We study the effect of using alternative metrics for measuring the reconstruction distance. We consider two popular metrics, Mean Squared Error (MSE) and Structural Similarity Index Measure (SSIM) (Wang et al., 2003), both of which have been widely used for image comparison (Zhang et al., 2019; Bhat et al., 2021; Saharia et al., 2022). We further observe that Xiao et al. (2021) uses features from a ResNet-50 pretrained with SimCLRv2 (Chen et al., 2020) on ImageNet, and achieves superior performance on CIFAR10 vs. CIFAR100. Thus, we also consider a SimCLRv2-based metric, in which we calculate the cosine distance between the SimCLRv2 features of the original and reconstructed images.

We present the performance of LMD under different distance metrics in Table 3. MSE and SSIM demonstrate poor performance when SVHN is the out-of-domain dataset. Our default choice LPIPS demonstrates strong and consistent performance, and attains the highest average ROC-AUC. SimCLRv2 is competitive but underperforms LPIPS. This suggests that deep feature based metrics are in general effective, and LPIPS is suitable as a default choice.

**Table 3:** ROC-AUC performance under different reconstruction distance metrics.

**ID**	**OOD**	**MSE**	**SSIM**	**LPIPS**	**SimCLRv2**
CIFAR10	CIFAR100	0.548	0.624	0.607	0.713
	SVHN	0.155	0.329	0.992	0.970
CIFAR100	CIFAR10	0.549	0.551	0.568	0.523
	SVHN	0.157	0.258	0.985	0.924
SVHN	CIFAR10	0.987	0.998	0.914	0.933
	CIFAR100	0.979	0.995	0.876	0.928
MNIST	KMNIST	0.998	0.997	0.984	0.983
	FashionMNIST	0.995	0.999	0.999	0.999
KMNIST	MNIST	0.835	0.922	0.978	0.920
	FashionMNIST	0.802	0.979	0.993	0.995
FashionMNIST	MNIST	0.993	0.960	0.992	0.961
	KMNIST	0.998	0.988	0.990	0.977
Average		0.750	0.800	**0.907**	0.902

LPIPS demonstrates consistent and robust results, while other metrics exhibit performance fluctuations. The bold values mean the best performance, i.e., the highest ROC-AUC, among the evaluated methods in each setting, where a setting is either an in-domain vs. out-of-domain dataset pair or the average across all dataset pairs.

#### 3.4.3 Number of reconstructions per image

**Figure 9:**
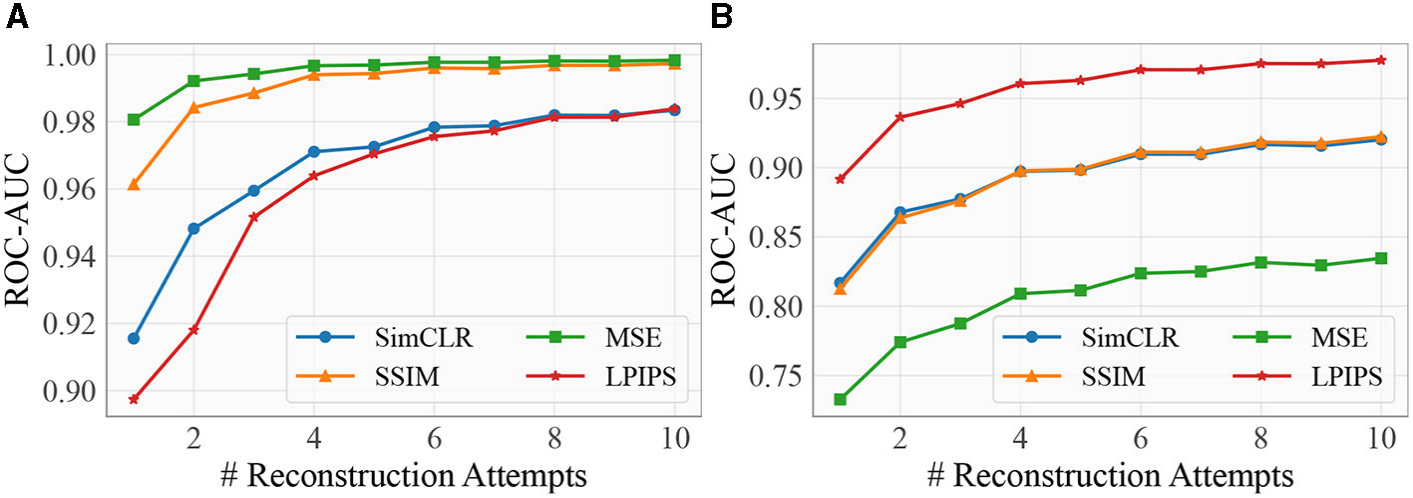
ROC-AUC vs. the number of reconstruction attempts. More reconstruction attempts enhances the OOD detection performance, irrespective of the distance metric. **(A)** MNIST vs. KMNIST. **(B)** KMNIST vs. MNIST.

We examine LMD's performance under different number of reconstructions per image. Figure 9 plots the ROC-AUC against the number of reconstructions per image for MNIST vs. KMNIST and KMNIST vs. MNIST. LMD's performance improves as the number of reconstructions increases, regardless of the choice of distance metric. The improvement is especially obvious for the first 5 attempts, and gradually plateaus as the number of attempts approaches 10. This suggests that it is generally sufficient to perform 10 attempts per image.

#### 3.4.4 Alternative instantiation of lifting and mapping

We observe that another intuitive way of lifting and mapping images with a diffusion model is to lift by diffusion to an intermediate step *t* in the noise schedule, and denoising back to the image distribution. We refer to this alternative instantiation as diffusion/denoising, and compare it with our default instantiation of masking/inpainting. Given that the image distribution is at *t* = 0 and the noise distribution is at *t* = *T*, the larger *t* we diffuse to, the further away we lift an image from the manifold. We consider different lifting distances with *t* = 250, *t* = 500, and *t* = 750, where the full schedule has *T* = 1000. We use our default alternating checkerboard 8 × 8 mask for masking/inpainting. We use 10 reconstructions per image and the LPIPS metric for both diffusion/denoising and masking/inpainting.

We present the performance in Table 4. Diffusion/denoising with *t* = 250 and *t* = 750 demonstrate suboptimal performance on several pairs, indicating that the lifting distance is too small or too large for the in-domain and out-of-domain to be distinguishable. *t* = 500 is competitive but underperforms masking/inpainting. This suggests that while LMD is robust to alternative choices of lifting and mapping, masking/inpainting is the recommended instantiation.

**Table 4:** ROC-AUC performance of using diffusion/denoising vs. masking/inpainting.

**ID**	**OOD**	**Denoising (*t* = 250)**	**Denoising (*t* = 500)**	**Denoising (*t* = 750)**	**Inpainting**
CIFAR10	CIFAR100	0.583	0.600	0.589	0.607
	SVHN	0.967	0.976	0.954	0.992
CIFAR100	CIFAR10	**0.568**	0.524	0.436	**0.568**
	SVHN	0.949	0.957	0.904	**0.985**
SVHN	CIFAR10	0.861	**0.966**	0.957	0.914
	CIFAR100	0.847	0.949	**0.957**	0.876
MNIST	KMNIST	0.956	**0.993**	0.715	0.984
	FashionMNIST	0.998	0.998	0.927	**0.999**
KMNIST	MNIST	0.645	0.972	0.721	**0.978**
	FashionMNIST	**0.998**	0.994	0.943	0.993
FashionMNIST	MNIST	0.428	0.941	0.876	**0.992**
	KMNIST	0.567	0.943	0.862	**0.990**
Average		0.781	0.901	0.820	**0.907**

Diffusion/denoising with *t* = 500 achieves reasonable performance but underperforms diffusion/inpainting. The bold values mean the best performance, i.e., the highest ROC-AUC, among the evaluated methods in each setting, where a setting is either an in-domain vs. out-of-domain dataset pair or the average across all dataset pairs.

#### 3.4.5 Alternative choices for the inpainting model

We perform qualitative evaluation on using other classes of inpainting models in the LMD framework. We consider **Masked Autoencoder (MAE)** (He et al., 2022) trained on CIFAR10,^5^ and **LaMa** (Suvorov et al., 2022),^6^ a GAN-based inpainting model, trained on CelebA-HQ. We perform *one* reconstruction per image, as both MAE and LaMa are deterministic.

**Figure 10:**
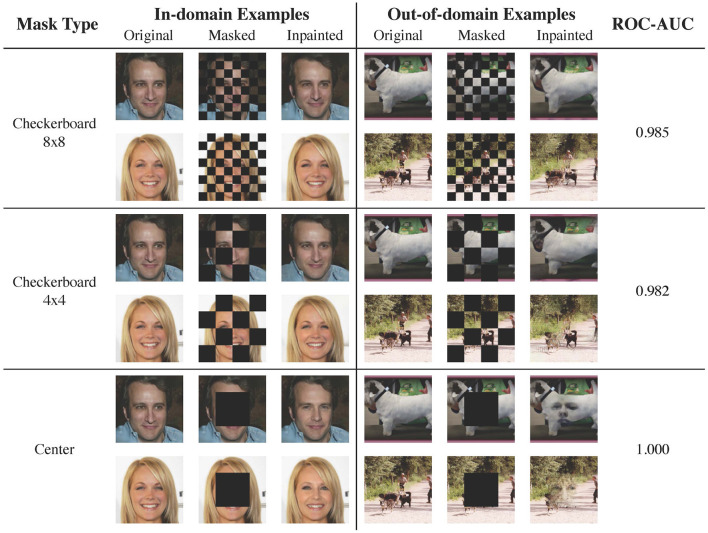
Reconstruction examples from CelebA-HQ (in-domain) and ImageNet (out-of-domain) using LaMa, a GAN-based inpainting model. Unlike the diffusion model, LaMa produces less visible artifacts. Even though it also introduces face-like artifacts with the center mask, the faces have the colors and textures of the surrounding unmasked regions.

Both models demonstrate lower performance than the diffusion model in various scenarios. Figure 10 shows LaMa's performance on CelebA-HQ vs. ImageNet. LaMa attains reasonable results, but it underperforms diffusion models. LaMa hallucinates faces with the center mask, but unlike the diffusion model, the color and texture of the hallucinated faces are very consistent with the surroundings.

**Figure 11:**
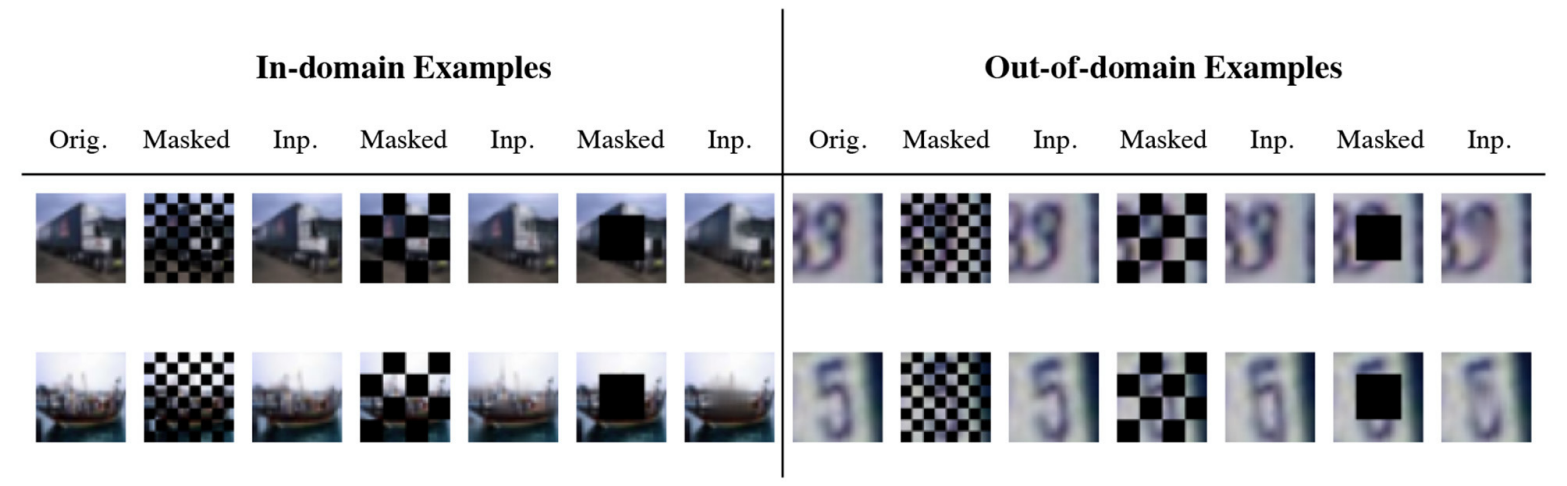
Reconstruction examples from CIFAR10 (in-domain) and SVHN (out-of-domain) using MAE. Differentiating between in-domain and out-of-domain inpaintings are hard, because reconstructing SVHN from only known regions is relatively simple, and because MAE is trained to have strong capability of inference from known regions.

Figure 11 shows MAE's performance on CIFAR10 vs. SVHN. Both in-domain and out-of-domain reconstructions are accurate when the individual masked patch sizes are small, while both deviate from the originals when the patch sizes are large. Performance-wise, inpainting with MAE only attains ROC-AUC 0.065 for checkerboard 8 × 8 mask, 0.178 for checkerboard 4 × 4 mask and 0.403 for center mask.

The suboptimal performance of alternative inpainting models can be attributed to their ability to leverage various sources of information—from not only its understanding of the training distribution, but also color or texture of unmasked parts of an image. Models like LaMa and MAE employ specialized loss functions and large masked ratios during training, and thus excel at inferring missing regions from known ones regardless of semantics. Consequently, these models are more prone to producing reasonable out-of-domain inpaintings, especially with simpler out-of-domain images. In contrast, a vanilla diffusion model is not specifically trained for inferring missing regions from the surroundings. It primarily relies on its understanding of the training distribution to perform inpainting, and thus attains robust performance.

## 4 Discussion

### 4.1 LMD's relationship with existing works

In the unsupervised setting, existing works generally follow one of the three paradigms: likelihood-based, reconstruction-based and feature-based. LMD is a reconstruction-based approach. Typically, reconstruction-based methods involve training a model using in-domain samples, and assessing the reconstruction quality of a test data point under the model. Prior works commonly use autoencoders (Sakurada and Yairi, 2014; Xia et al., 2015; Zhou and Paffenroth, 2017; Zong et al., 2018) or GANs (Schlegl et al., 2017; Li et al., 2018). One concurrent work (Graham et al., 2022) utilizes diffusion models, and considers image reconstructions under varying numbers of diffusion and denoising steps. This contrasts with LMD, which repeatedly performs masking and inpainting with fixed number of steps. These two approaches are orthogonal and complementary.

The likelihood-based paradigm has been extensively explored, with early contributions dating back to Bishop (1994). The core idea is to approximate the in-domain distribution with a generative model that has likelihood computation capability (Salimans et al., 2017; Kingma and Dhariwal, 2018). Intuitively, the model should assign higher likelihood to in-domain data than out-of-domain data, but various studies have observed that such assumption often does not hold (Choi et al., 2018; Nalisnick et al., 2018; Kirichenko et al., 2020). One line of work addresses this issue under a typicality test framework (Ren et al., 2019; Serrà et al., 2019; Xiao et al., 2020). Essentially, they view likelihood as a model statistic rather than a literal measure of how likely a data point is in-domain. They examine the extent to which the model statistic of a test data point deviates from the typical distribution of model statistics for in-domain data. Notably, this is complementary to LMD, as the reconstruction distance can also be viewed as a model statistic. Other likelihood-based approaches include adjusting the likelihood by background likelihood (Ren et al., 2019), image complexity (Serrà et al., 2019) or the likelihood under optimal model configurations (Xiao et al., 2020), or improving the generative model architectures (Maaløe et al., 2019; Kirichenko et al., 2020).

The feature-based paradigm usually involves extracting lower-dimensional features from the data from unsupervised sources, such as autoencoders (Denouden et al., 2018), generative models (Ahmadian and Lindsten, 2021), self-supervised training (Hendrycks et al., 2019; Bergman and Hoshen, 2020; Tack et al., 2020; Sehwag et al., 2021) or pretrained feature extractors (Xiao et al., 2021). They then perform detection in lower-dimensional space, typically with simple techniques like fitting one-class Support Vector Machines or Gaussian Mixture Models.

### 4.2 Limitation and future work

One limitation of LMD is the speed. Vanilla diffusion models have a time-consuming denoising process that involves a large number of sampling steps. Therefore, similar to other diffusion-based approaches for various tasks (Meng et al., 2021; Lugmayr et al., 2022; Saharia et al., 2022), LMD is currently not well-suited for real-time OOD detection. Several recent works have proposed methods to accelerate the sampling process of pre-trained diffusion models through noise rescaling (Nichol and Dhariwal, 2021), sampler optimization (Watson et al., 2022), or numerical methods (Liu et al., 2022; Wizadwongsa and Suwajanakorn, 2023). One future direction is to harness these methods to expedite LMD's detection.

Another potential extension is to utilize more advanced methods for aggregating reconstruction distances from multiple reconstructions, or even under different masks or distance metrics. As briefly discussed in Section 4.1, this can involve integrating typicality test approaches such as multiple hypothesis testing or learning density models (Nalisnick et al., 2019; Morningstar et al., 2021; Bergamin et al., 2022).

## 5 Conclusion

We propose a novel method, *Lift, Map, Detect* (LMD), for unsupervised out-of-distribution detection. LMD leverages the diffusion model's strong ability in mapping images onto its training manifold, and detects images with large distance between the original and mapped images as OOD. Our extensive experiments and analysis show that LMD achives strong performance for various image distributions with different characteristics. Some future directions of improvement include accelerating LMD's speed and leveraging advanced aggregation for reconstruction distance.

## Data availability statement

Publicly available datasets were analyzed in this study. This data can be found here: CIFAR10, CIFAR100, SVHN, MNIST, KMNIST, and FashionMNIST: can be accessed through https://pytorch.org/vision/stable/datasets.html. CelebA-HQ: https://github.com/tkarras/progressive_growing_of_gans. ImageNet: https://www.image-net.org/. LSUN bedroom and LSUN classroom: https://github.com/fyu/lsun.

## Ethics statement

Written informed consent was not obtained from the individual(s) for the publication of any potentially identifiable images or data included in this article, because these human face images are either from the public datasets CelebA-HQ and FFHQ, which are widely used in the machine learning and computer vision communities, or synthetic faces created by generative models.

## Author contributions

ZL and JZ contributed to the design of the research, performed the experiments, and wrote the manuscript. KW is the PhD supervisor of ZL and JZ, he conceptualized and directed the research, and revised the manuscript. All authors approved the submitted version.

## Footnotes

^1^We use the implementation of https://github.com/richzhang/PerceptualSimilarity.

^2^ https://github.com/yang-song/score_sde_pytorch

^3^ https://github.com/XavierXiao/Likelihood-Regret

^4^ https://github.com/microsoft/SimMIM

^5^ https://github.com/IcarusWizard/MAE

^6^ https://github.com/advimman/lama

## References

[B1] Ahmadian A. Lindsten F. (2021). “Likelihood-free out-of-distribution detection with invertible generative models,” in IJCAI, 2119–2125. 10.24963/ijcai.2021/292

[B2] Alaluf Y. Patashnik O. Cohen-Or D. (2021). “Restyle: a residual-based stylegan encoder via iterative refinement,” in Proceedings of the IEEE/CVF International Conference on Computer Vision, 6711–6720. 10.1109/ICCV48922.2021.00664

[B3] Bergamin F. Mattei P.-A. Havtorn J. D. Senetaire H. Schmutz H. Maalœ L.. (2022). “Model-agnostic out-of-distribution detection using combined statistical tests,” in International Conference on Artificial Intelligence and Statistics (PMLR), 10753-10776.

[B4] Bergman L. Hoshen Y. (2020). Classification-based anomaly detection for general data. arXiv preprint arXiv:2005.02359.

[B5] Bhat S. F. Alhashim I. Wonka P. (2021). “Adabins: depth estimation using adaptive bins,” in Proceedings of the IEEE/CVF Conference on Computer Vision and Pattern Recognition, 4009–4018.

[B6] Bishop C. M. (1994). Novelty detection and neural network validation. IEE Proc. Vision Image Sig. Proc. 141, 217–222. 10.1049/ip-vis:19941330

[B7] Chen T. Kornblith S. Swersky K. Norouzi M. Hinton G. E. (2020). “Big self-supervised models are strong semi-supervised learners,” in Advances in Neural Information Processing Systems, 22243–22255.

[B8] Choi H. Jang E. Alemi A. A. (2018). Waic, but why? Generative ensembles for robust anomaly detection. arXiv preprint arXiv:1810.01392.

[B9] Clanuwat T. Bober-Irizar M. Kitamoto A. Lamb A. Yamamoto K. Ha D. (2018). Deep learning for classical Japanese literature. arXiv preprint arXiv:1812.01718.32828440

[B10] Denouden T. Salay R. Czarnecki K. Abdelzad V. Phan B. Vernekar S. (2018). Improving reconstruction autoencoder out-of-distribution detection with mahalanobis distance. arXiv preprint arXiv:1812.02765.

[B11] Gong Y. Liao P. Zhang X. Zhang L. Chen G. Zhu K.. (2021). Enlighten-gan for super resolution reconstruction in mid-resolution remote sensing images. Rem. Sens. 13:1104. 10.3390/rs13061104

[B12] Graham M. S. Pinaya W. H. Tudosiu P.-D. Nachev P. Ourselin S. Cardoso M. J. (2022). Denoising diffusion models for out-of-distribution detection. arXiv preprint arXiv:2211.07740. 10.1109/CVPRW59228.2023.0029638228075

[B13] Hamet P. Tremblay J. (2017). Artificial intelligence in medicine. Metabolism 69, S36–S40. 10.1016/j.metabol.2017.01.011 28126242

[B14] He K. Chen X. Xie S. Li Y. Dollár P. Girshick R. (2022). “Masked autoencoders are scalable vision learners,” in Proceedings of the IEEE/CVF Conference on Computer Vision and Pattern Recognition, 16000–16009. 10.1109/CVPR52688.2022.01553

[B15] Hendrycks D. Gimpel K. (2016). A baseline for detecting misclassified and out-of-distribution examples in neural networks. arXiv preprint arXiv:1610.02136.38090830

[B16] Hendrycks D. Mazeika M. Dietterich T. (2018). Deep anomaly detection with outlier exposure. arXiv preprint arXiv:1812.04606.

[B17] Hendrycks D. Mazeika M. Kadavath S. Song D. (2019). “Using self-supervised learning can improve model robustness and uncertainty,” in Advances in Neural Information Processing Systems 32.

[B18] Ho J. Jain A. Abbeel P. (2020). “Denoising diffusion probabilistic models,” in Advances in Neural Information Processing Systems, 6840–6851.

[B19] Huang R. Geng A. Li Y. (2021). “On the importance of gradients for detecting distributional shifts in the wild,” in Advances in Neural Information Processing Systems, 677–689.

[B20] Karras T. Aila T. Laine S. Lehtinen J. (2017). Progressive growing of gans for improved quality, stability, and variation. CoRR, abs/1710.10196.

[B21] Karras T. Laine S. Aila T. (2019). “A style-based generator architecture for generative adversarial networks,” in Proceedings of the IEEE/CVF Conference on Computer Vision and Pattern Recognition, 4401–4410. 10.1109/CVPR.2019.00453 32012000

[B22] Kingma D. P. Dhariwal P. (2018). “Glow: generative flow with invertible 1x1 convolutions,” in Advances in Neural Information Processing Systems 31.

[B23] Kirichenko P. Izmailov P. Wilson A. G. (2020). “Why normalizing flows fail to detect out-of-distribution data,” in Advances in Neural Information Processing Systems, 20578–20589.

[B24] Krizhevsky A. (2009). Learning multiple layers of features from tiny images. Technical report.

[B25] Krizhevsky A. Sutskever I. Hinton G. E. (2012). “Imagenet classification with deep convolutional neural networks,” in Advances in Neural Information Processing Systems 25.

[B26] LeCun Y. Cortes C. Burges C. (2010). Mnist handwritten digit database. ATT Labs. Available online at: http://yann.lecun.com/exdb/mnist (accessed July 08, 2023).

[B27] Lee K. Lee K. Lee H. Shin J. (2018). “A simple unified framework for detecting out-of-distribution samples and adversarial attacks,” in Advances in Neural Information Processing Systems 31.

[B28] Li D. Chen D. Goh J. Ng S.- K. (2018). Anomaly detection with generative adversarial networks for multivariate time series. arXiv preprint arXiv:1809.04758.

[B29] Liang S. Li Y. Srikant R. (2017). Enhancing the reliability of out-of-distribution image detection in neural networks. arXiv preprint arXiv:1706.02690.

[B30] Liu L. Ren Y. Lin Z. Zhao Z. (2022). Pseudo numerical methods for diffusion models on manifolds. arXiv preprint arXiv:2202.09778.

[B31] Lugmayr A. Danelljan M. Romero A. Yu F. Timofte R. Van Gool L. (2022). “Repaint: inpainting using denoising diffusion probabilistic models,” in Proceedings of the IEEE/CVF Conference on Computer Vision and Pattern Recognition, 11461–11471. 10.1109/CVPR52688.2022.01117

[B32] Maalœ L. Fraccaro M. Liévin V. Winther O. (2019). “Biva: a very deep hierarchy of latent variables for generative modeling,” in Advances in Neural Information Processing Systems 32.

[B33] Meng C. He Y. Song Y. Song J. Wu J. Zhu J.-Y.. (2021). “Sdedit: guided image synthesis and editing with stochastic differential equations,” in International Conference on Learning Representations.

[B34] Morningstar W. Ham C. Gallagher A. Lakshminarayanan B. Alemi A. Dillon J. (2021). “Density of states estimation for out of distribution detection,” in International Conference on Artificial Intelligence and Statistics (PMLR), 3232–3240.

[B35] Nalisnick E. Matsukawa A. Teh Y. W. Gorur D. Lakshminarayanan B. (2018). Do deep generative models know what they don't know? *arXiv preprint arXiv:1810.09136*.

[B36] Nalisnick E. T. Matsukawa A. Teh Y. W. Lakshminarayanan B. (2019). Detecting out-of-distribution inputs to deep generative models using a test for typicality. arXiv preprint arXiv:1906.02994.

[B37] Netzer Y. Wang T. Coates A. Bissacco A. Wu B. Ng A. Y. (2011). “Reading digits in natural images with unsupervised feature learning,” in NIPS Workshop on Deep Learning and Unsupervised Feature Learning, 7.

[B38] Nichol A. Q. Dhariwal P. (2021). “Improved denoising diffusion probabilistic models,” in International Conference on Machine Learning (PMLR), 8162–8171.

[B39] Ren J. Liu P. J. Fertig E. Snoek J. Poplin R. Depristo M.. (2019). “Likelihood ratios for out-of-distribution detection,” in Advances in Neural Information Processing Systems 32.

[B40] Rigano C. (2019). Using artificial intelligence to address criminal justice needs. Natl. Inst. Justice J. 280, 1–10.36871544

[B41] Ruff L. Vandermeulen R. A. Görnitz N. Binder A. Müller E. Müller K.-R.. (2019). Deep semi-supervised anomaly detection. arXiv preprint arXiv:1906.02694.

[B42] Russakovsky O. Deng J. Su H. Krause J. Satheesh S. Ma S.. (2015). ImageNet large scale visual recognition challenge. Int. J. Comput. Vis. 115, 211–252. 10.1007/s11263-015-0816-y

[B43] Saharia C. Ho J. Chan W. Salimans T. Fleet D. J. Norouzi M. (2022). Image super-resolution via iterative refinement. IEEE Trans. Patt. Analy. Mach. Intell. 45, 4713–4726. 10.1109/TPAMI.2022.3204461 36094974

[B44] Sakurada M. Yairi T. (2014). “Anomaly detection using autoencoders with nonlinear dimensionality reduction,” in Proceedings of the MLSDA 2014 2nd Workshop on Machine Learning for Sensory Data Analysis, 4–11. 10.1145/2689746.2689747

[B45] Salimans T. Karpathy A. Chen X. Kingma D. P. (2017). Pixelcnn++: improving the pixelcnn with discretized logistic mixture likelihood and other modifications. arXiv preprint arXiv:1701.05517.

[B46] Schlegl T. Seeböck P. Waldstein S. M. Schmidt-Erfurth U. Langs G. (2017). “Unsupervised anomaly detection with generative adversarial networks to guide marker discovery,” in International Conference on Information Processing in Medical Imaging (Springer), 146–157. 10.1007/978-3-319-59050-9_12

[B47] Sehwag V. Chiang M. Mittal P. (2021). Ssd: A unified framework for self-supervised outlier detection. arXiv preprint arXiv:2103.12051.

[B48] Serrà J. Álvarez D. Gómez V. Slizovskaia O. Nú nez J. F. Luque J. (2019). Input complexity and out-of-distribution detection with likelihood-based generative models. arXiv preprint arXiv:1909.11480.

[B49] Sohl-Dickstein J. Weiss E. Maheswaranathan N. Ganguli S. (2015). “Deep unsupervised learning using nonequilibrium thermodynamics,” in International Conference on Machine Learning (PMLR), 2256–2265.

[B50] Song Y. Ermon S. (2019). “Generative modeling by estimating gradients of the data distribution,” in Advances in Neural Information Processing Systems 32.

[B51] Song Y. Sohl-Dickstein J. Kingma D. P. Kumar A. Ermon S. Poole B. (2020). Score-based generative modeling through stochastic differential equations. arXiv preprint arXiv:2011.13456.

[B52] Suvorov R. Logacheva E. Mashikhin A. Remizova A. Ashukha A. Silvestrov A.. (2022). “Resolution-robust large mask inpainting with fourier convolutions,” in Proceedings of the IEEE/CVF Winter Conference on Applications of Computer Vision, 2149–2159. 10.1109/WACV51458.2022.0032337235458

[B53] Tack J. Mo S. Jeong J. Shin J. (2020). “CSI: novelty detection via contrastive learning on distributionally shifted instances,” in Advances in Neural Information Processing Systems 11839–11852.

[B54] Toda R. Teramoto A. Kondo M. Imaizumi K. Saito K. Fujita H. (2022). Lung cancer ct image generation from a free-form sketch using style-based pix2pix for data augmentation. Sci. Rep. 12:12867. 10.1038/s41598-022-16861-5 35896575 PMC9329467

[B55] Wang H. Li Z. Feng L. Zhang W. (2022). “Vim: out-of-distribution with virtual-logit matching,” in Proceedings of the IEEE/CVF Conference on Computer Vision and Pattern Recognition, 4921–4930. 10.1109/CVPR52688.2022.00487

[B56] Wang Z. Simoncelli E. P. Bovik A. C. (2003). “Multiscale structural similarity for image quality assessment,” in The Thrity-Seventh Asilomar Conference on Signals, Systems and Computers (IEEE), 1398–1402.

[B57] Watson D. Chan W. Ho J. Norouzi M. (2022). “Learning fast samplers for diffusion models by differentiating through sample quality,” in International Conference on Learning Representations.

[B58] Wizadwongsa S. Suwajanakorn S. (2023). Accelerating guided diffusion sampling with splitting numerical methods. arXiv preprint arXiv:2301.11558.

[B59] Xia Y. Cao X. Wen F. Hua G. Sun J. (2015). “Learning discriminative reconstructions for unsupervised outlier removal,” in Proceedings of the IEEE International Conference on Computer Vision, 1511–1519. 10.1109/ICCV.2015.177

[B60] Xiao H. Rasul K. Vollgraf R. (2017). Fashion-mnist: a novel image dataset for benchmarking machine learning algorithms. CoRR, abs/1708.07747.

[B61] Xiao Z. Yan Q. Amit Y. (2020). “Likelihood regret: an out-of-distribution detection score for variational auto-encoder,” in Advances in Neural Information Processing Systems, 20685–20696.

[B62] Xiao Z. Yan Q. Amit Y. (2021). Do we really need to learn representations from in-domain data for outlier detection? *arXiv preprint arXiv:2105.09270*.

[B63] Xie Z. Zhang Z. Cao Y. Lin Y. Bao J. Yao Z.. (2022). “Simmim: a simple framework for masked image modeling,” in Proceedings of the IEEE/CVF Conference on Computer Vision and Pattern Recognition, 9653–9663. 10.1109/CVPR52688.2022.00943

[B64] Yang L. Zhang Z. Song Y. Hong S. Xu R. Zhao Y.. (2022). Diffusion models: a comprehensive survey of methods and applications. arXiv preprint arXiv:2209.00796.

[B65] Yu F. Zhang Y. Song S. Seff A. Xiao J. (2015). Lsun: construction of a large-scale image dataset using deep learning with humans in the loop. arXiv preprint arXiv:1506.03365.

[B66] Zhang K. A. Cuesta-Infante A. Xu L. Veeramachaneni K. (2019). Steganogan: high capacity image steganography with gans. arXiv preprint arXiv:1901.03892.

[B67] Zhang R. Isola P. Efros A. A. Shechtman E. Wang O. (2018). “The unreasonable effectiveness of deep features as a perceptual metric,” in Proceedings of the IEEE Conference on Computer Vision and Pattern Recognition, 586–595. 10.1109/CVPR.2018.00068

[B68] Zhou C. Paffenroth R. C. (2017). “Anomaly detection with robust deep autoencoders,” in Proceedings of the 23rd ACM SIGKDD International Conference on Knowledge Discovery and Data Mining, 665–674. 10.1145/3097983.3098052

[B69] Zong B. Song Q. Min M. R. Cheng W. Lumezanu C. Cho D.. (2018). “Deep autoencoding gaussian mixture model for unsupervised anomaly detection,” in International Conference on Learning Representations.

